# Restoration of physiologic loading modulates engineered intervertebral disc structure and function in an in vivo model

**DOI:** 10.1002/jsp2.1086

**Published:** 2020-05-13

**Authors:** Sarah E. Gullbrand, Dong Hwa Kim, Beth G. Ashinsky, Edward D. Bonnevie, Harvey E. Smith, Robert L. Mauck

**Affiliations:** ^1^ Translational Musculoskeletal Research Center Corporal Michael J. Crescenz VA Medical Center Philadelphia Pennsylvania USA; ^2^ McKay Orthopaedic Research Laboratory, Department of Orthopaedic Surgery University of Pennsylvania Philadelphia Pennsylvania USA; ^3^ School of Biomedical Engineering, Science and Health Systems Drexel University Philadelphia Pennsylvania USA; ^4^ Department of Bioengineering University of Pennsylvania Philadelphia Pennsylvania USA

**Keywords:** integration, physiologic loading, rat model, tissue engineering

## Abstract

Tissue‐engineered whole disc replacements are an emerging treatment strategy for advanced intervertebral disc degeneration. A challenge facing the translation of tissue‐engineered disc replacement to clinical use are the opposing needs of initial immobilization to advantage integration contrasted with physiologic loading and its anabolic effects. Here, we utilize our established rat tail model of tissue engineered disc replacement with external fixation to study the effects of remobilization at two time points postimplantation on engineered disc structure, composition, and function. Our results suggest that the restoration of mechanical loading following immobilization enhanced collagen and proteoglycan content within the nucleus pulposus and annulus fibrosus of the engineered discs, in addition to improving the integration of the endplate region of the construct with native bone. Despite these benefits, angulation of the vertebral bodies at the implanted level occurred following remobilization at both early and late time points, reducing tensile failure properties in the remobilized groups compared to the fixed group. These results demonstrate the necessity of restoring physiologic mechanical loading to engineered disc implants in vivo, and the need to transition toward their evaluation in larger animal models with more human‐like anatomy and motion compared to the rat tail.

## Graphical abstract

Here, we utilize our established rat tail model of tissue engineered disc replacement with external fixation to study the effects of remobilization at two time points post‐implantation on engineered disc structure, composition and function. We demonstrate that restoration of mechanical loading enhanced engineered disc composition, but compromised motion segment morphology and integration strength. These findings highlight the need to transition to larger animal models with more human‐like anatomy and motion compared to the rat tail to study the effects of in vivo loading on tissue engineered discs.

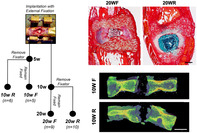

## 1 INTRODUCTION

Intervertebral disc degeneration manifests as a spectrum of compositional, structural, and functional changes to the disc and its subcomponents, the annulus fibrosus (AF), and nucleus pulposus (NP).^1^ Disc degeneration is frequently associated with axial back pain and neurogenic extremity pain, thereby necessitating clinical intervention.^2^ Severe degeneration is commonly treated with spinal fusion surgery, which removes the degenerative disc, decompresses the adjacent nerve roots, and immobilizes the pathologic motion segment.^3^ Fusion does not restore native disc structure or function, and has been associated with degeneration of adjacent motion segments, potentially via alterations to whole spine kinematics.^4^ Due to the limitations of currently available clinical treatments, new strategies that aim to functionally regenerate the disc have become a major area of research. For end‐stage degeneration, where all disc substructures have undergone degenerative changes, a biologic tissue‐engineered disc replacement, which could integrate with the surrounding native tissues, restore healthy disc structure and function, and continually remodel during physiologic use, is an attractive treatment option.^5^

Tissue‐engineered constructs for whole disc replacement generally consist of an isotropic cell‐seeded hydrogel for the NP region, surrounded by a cell‐seeded biomaterial for the AF region. This AF region may further be refined to recapitulate aspects of the native AF anisotropy and organization, with recent formulations employing circumferentially oriented collagen and ordered angle‐ply laminate structures.^5^, ^6^ While a variety of such whole, tissue engineered disc designs have been characterized in vitro, the in vivo evaluation of such constructs has been relatively limited to date.^7^, ^8^, ^9^, ^10^, ^11^, ^12^, ^13^ An inherent challenge in the process of in vivo translation of these tissue engineered disc replacements is the balance between initially protecting and stabilizing the implant to allow for sufficient integration to retain the construct in the disc space, but eventually providing for the restoration of physiologic loading to maintain and promote disc homeostasis through mechanical loading.^14^, ^15^ Additionally, the degree to which the engineered implant matches disc functional properties at the time of implantation (vs matures in situ) will define the degree to which it can bear load from the outset. The first tissue‐engineered discs evaluated in vivo were composed of an NP cell‐seeded alginate hydrogel surrounded by an AF cell‐contracted aligned collagen gel, and were implanted for up to 8 months in the rat caudal disc space without fixation.^7^ While these constructs were retained and performed well in the rat caudal spine, when translated to a larger canine cervical disc replacement model, displacement of the implant occurred in nearly half the animals investigated.^16^ It is not clear if this displacement was due to the larger loads placed on the implant, or whether it was due the greater degrees of freedom in motion about the cervical spine compared to the lumbar spine, or both.

Our group has developed a whole tissue engineered disc‐like angle ply (DAPS) construct composed of a cell‐seeded hyaluronic acid hydrogel for the NP region, surrounded by cell‐seeded layers of electrospun poly (ε‐caprolactone) (PCL) where the nanofibers within a layer are oriented at ±30° to the long axis of the construct to mimic the hierarchical structure of the native AF.^8^ These tissue‐engineered discs can be fabricated with NP and AF components only (DAPS), or can include acellular PCL foams as endplate analogs (eDAPS).^9^, ^10^ We previously demonstrated that for DAPS implanted in vivo in the rat caudal spine to remain in place, the motion segment had to be immobilized using an external fixation device to prevent extrusion.^17^ However, long‐term immobilization of the native intervertebral disc results in substantial alterations to disc height and mechanical properties indicative of degeneration, in addition to reduced disc cell anabolism and increased catabolism.^18^, ^19^, ^20^, ^21^ Long‐term immobilization is therefore not only contrary to the goals of a tissue engineered disc replacement, but also would likely have a detrimental effect on engineered disc health.

Thus, the purpose of this study was to determine the effects of removal of external fixation (remobilization) on engineered disc composition, structure, and mechanical function, when the immobilization was removed at different times postimplantation. We hypothesized that after sufficient time of implantation to allow for boney fixation, restoration of physiologic mechanical loading would improve engineered disc composition and mechanical properties.

## 2 METHODS

### 2.1 Study design

**FIGURE 1:**
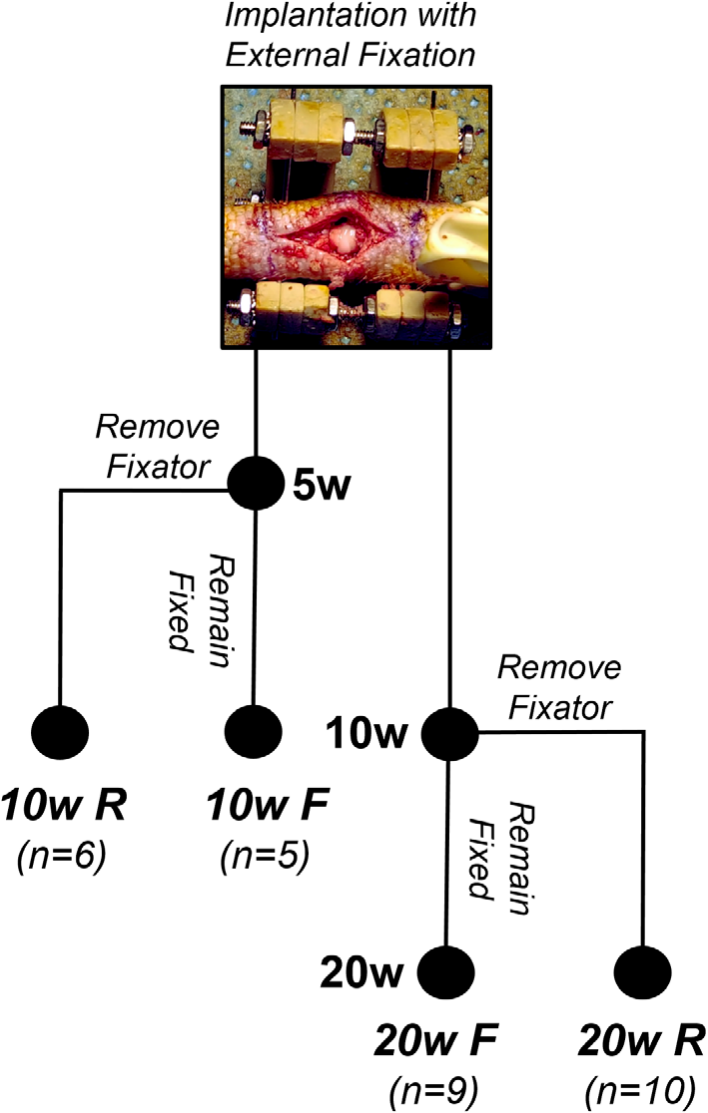
Schematic illustrating the study design and time points for remobilization

The effects of remobilization (removal of external fixation) on eDAPS structure, composition, and function were assessed via the study design summarized in Figure 1. Thirty athymic rats underwent a surgical procedure to implant precultured eDAPS within the caudal spine and immobilize the implanted motion segment with an external fixator. Both early (5 weeks postimplantation) and late (10 weeks postimplantation) remobilization time points were investigated in this study. To do so, one subset of animals was designated for a 10‐week study endpoint, where the external fixator was removed in 6 rats at 5 weeks postimplantation (10 W R), but left in place in the remaining 5 rats for the 10 week study duration (10 W F). A second subset of animals was designated for a 20‐week study endpoint, where the external fixator was removed in 10 rats at 10 weeks postimplantation (20 W R), but left in place in 9 rats for the 20 week study duration (20 W F). At the study endpoints, animals were euthanized and vertebral body‐eDAPS‐vertebral body motion segments and adjacent native control motion segments were harvested for analysis. All motion segments in each experimental group underwent MRI T2 mapping, and were subsequently split for histological analysis (n = 2‐3 per group), or mechanical testing followed by biochemical assays (n = 3‐7 per group). A subset of the data generated for the 10 W F and 20 W F groups has been published previously in Gullbrand *et al*, and were utilized as controls for this study.^10^

### 2.2 eDAPS fabrication and preculture

Bovine NP and AF cells were isolated from caudal discs (~3 years old, ~2 hours after sacrifice; JBS Souderton, Inc. Souderton, PA), as previously described.^22^ Cells were expanded to passage two prior to use in Basal Media containing Dulbecco's Modified Eagle Medium (DMEM; Gibco, Invitrogen Life Sciences, Carlsbad, CA), 10% fetal bovine serum (Gibco), and 1% Antibiotic‐Antimycotic (Gibco).

eDAPS sized for the rat caudal spine (5 mm high, 4‐5 mm diameter) were fabricated, cultured and seeded according to our previously established methods.^9^, ^23^ Briefly, the AF region of the eDAPS was fabricated by concentrically wrapping electrospun scaffolds of layered PCL and poly(ethylene oxide) (PEO) nanofibers to achieve a layered structure 4‐5 mm in diameter and 2 mm in height, with fiber angles at ±30° relative to the eDAPS long axis. The PEO fibers were subsequently removed during the process of scaffold sterilization and rehydration. The AF scaffolds were coated in 20 μg/mL of fibronectin (Sigma‐Aldrich) overnight, and bovine AF cells were seeded on the top and bottom side of the AF region (1 × 10^6^ cells per side). For the NP region of the eDAPS, bovine NP cells (20 million cells/mL) were suspended in 1% w/v methyacrylated hyaluronic acid (MeHA), dissolved in 0.05% photoinitiator (Irgacure 2959, Ciba‐Geigy).^22^, ^24^ The MeHA hydrogel was UV cured for 10 minutes between two glass plates, and punched to yield constructs 2 mm in diameter and 1.5 mm in height. The endplate region of the eDAPS consists of a porous PCL foam, fabricated via a salt leaching process and punched to create acellular constructs 4 mm in diameter and 1.5 mm in height.^10^, ^25^ In a subset of eDAPS utilized in animals with a 20 week study endpoint, zirconia nanoparticles were incorporated within the PCL foams to render them radiopaque.^26^

The cell‐seeded AF and NP regions of the eDAPS were cultured separately for 2 weeks in chemically defined media, composed of high glucose DMEM supplemented with 1% PSF, 40 ng/mL dexamethasone (Sigma‐Aldrich), 50 μg/mL ascorbate 2‐phosphate (Sigma‐Aldrich), 40 μg/mL L‐proline (Sigma‐Aldrich), 100 μg/mL sodium pyruvate (Corning Life Sciences), 0.1% insulin, transferrin, and selenious acid (ITS Premix Universal Culture Supplement; Corning), 1.25 mg/mL bovine serum albumin (Sigma‐Aldrich), 5.35 μg/mL linoleic acid (Sigma‐Aldrich), and 10 ng/mL TGF‐β3 (R&D Systems).^23^ The AF and NP regions were combined after 2 weeks, and two acellular PCL foam endplates were apposed to either side of the AF region using 31G needles.^25^, ^27^ Media were changed three time per week, and the needles were removed immediately prior to implantation.

### 2.3 eDAPS implantation and remobilization

All in vivo studies were approved by the Corporal Michael J. Crescenz Veterans Affairs Medical Center Institutional Animal Care and Use Committee, and were performed according to the guidelines recommended by this committee. Following 5 weeks of preculture, eDAPS were implanted in the C8‐C9 disc space of the athymic rat caudal spine (Foxn1^rnu^ retired breeders, Envigo), as previously described.^9^, ^10^ Under anesthesia and using standard aseptic techniques, two kirschner wires were passed through the C8 and C9 vertebral bodies, allowing for the placement of an external fixator designed to immobilize the implanted level. The native disc and portion of the vertebral endplates were removed using a high speed burr, and the eDAPS were press fit into the evacuated space. The incision was closed with suture, and the rats were returned to normal cage activity. As per Figure 1, at 5 or 10 weeks postimplantation, a subset of animals were anesthetized, and the external fixator and kirschner wires were removed (10 W R, 20 W R groups) to restore physiologic loading to the eDAPS implant. The external fixator was left in place in the remaining animals (10 W F, 20 W F groups). After recovery from anesthesia, the rats were returned to normal cage activity for the remainder of the study.

### 2.4 Radiographic analysis and magnetic resonance imaging

eDAPS implanted motion segments from the 10 W R and 10 W F groups underwent μCT scans (Scanco μCT50, 10 μm resolution) to assess bone morphology and vertebral anatomic positioning. μCT scans were performed on isolated motion segments following euthanasia and immediate fixator removal in the 10 W F groups. The angle between the vertebral bodies adjacent to the eDAPS was measured from mid‐coronal μCT slices, using ImageJ. In the 20 W R and 20 W F groups, serial radiographs of the same animal were taken using a fluoroscope over the duration of the study in order to capture dynamic changes in vertebral body angulation over time. Awake animals were briefly restrained for fluoroscopy, and the animals were positioned in the same orientation as for the eDAPS implantation surgery. Radiographs in the fixed group were obtained with the fixator in place. Radiographs were obtained immediately postoperative, and at 10 weeks (immediately following fixator removal in the remobilized group), 15 weeks and 20 weeks. From these images, the angle between vertebral bodies was quantified in Image J.

After euthanasia, eDAPS implanted and native rat tail motion segments underwent MRI scanning at 4.7 T using a multi‐echo‐multi‐spin sequence for T2 mapping (0.5 mm slice thickness, 117 μm in plane resolution, 16 echoes, TR/TE = 2000/11.13 ms), and a custom 17 mm diameter solenoid coil.^28^ From the image series, average T2 maps for each experimental group were generated using a custom MATLAB code, as previously described, to quantify T2 relaxation times in the NP.^29^

### 2.5 Biomechanical testing and analysis

eDAPS implanted and native motion segments were subjected to compressive mechanical testing, followed by tension to failure testing, as previously described.^10^ The skin of the tail was removed and the vertebral bodies adjacent to the eDAPS were cleared of soft tissue. Ink marks were placed on the vertebral bone immediately distal and proximal to the eDAPS to allow for optical displacement tracking during testing. Motion segments were potted in a low melting temperature indium casting alloy, and subjected to a testing protocol consisting of 20 cycles of compression from 0 to −3 N (0 to −0.25 MPa) at 0.05 Hz (Instron 5948) in a bath of phosphate‐buffered saline at room temperature. The 20th cycle of compression was used to calculate toe and linear region modulus, and transition and maximum strain. After compression testing, a complete circumferential dissection of the muscle and tendon surrounding the eDAPS was performed, and tension to failure testing was performed at 0.025 mm/s. A bi‐linear fit of the tension curves was used to quantify toe and linear tensile modulus and failure stress and strain. Mechanical properties in tension and compression were normalized to disc height and area obtained from MR images assuming circular geometry of the eDAPS.

### 2.6 Evaluation of eDAPS structure and composition

Implanted eDAPS subjected to mechanical testing were removed from the motion segment and were manually dissected into NP, AF, and EP portions and individually digested overnight in proteinase K at 60°C. Collagen content of each region was quantified using the p‐diaminobenzaldehyde/chloramine‐T assay for ortho‐hydroxyproline, and glycosaminoglycan (GAG) content was quantified using the dimethylmethylene blue (DMMB) dye binding assay.^30^, ^31^ Collagen and GAG content were normalized to sample wet weight, and compared to the composition of eDAPS prior to implantation (5 weeks of preculture).

Additional samples from each experimental group were fixed in 10% neutral buffered formalin, decalcified (Formical‐2000, Decal Chemical Corporation, Tallman, NY) and processed through paraffin for histological analysis. Sections of 10 μm were stained with Alcian blue (GAGs) and picrosirius red (collagen), or with the Mallory‐Heidenhain trichrome stain (orange = erythrocytes, pink = mineralized collagen, blue = unmineralized collagen), and imaged under brightfield. Second harmonic generation (SHG) images of the Alcian blue and picrosirius red‐stained sections were also obtained on a Nikon A1R multiphoton microscope equipped with a Spectra Physics Deep See Insight tunable laser set to 880 nm. Z‐stacks of 0.4 μm thickness were captured and presented as an average intensity projection.

For immunohistochemistry, sections were rehydrated and serially incubated at room temperature in proteinase K (Dako) for 5 minutes, 3% hydrogen peroxide for 10 minutes, and horse serum for 30 minutes (Vectastain ABC Universal Kit, Vector Laboratories). This was followed by overnight incubation at 4°C with primary antibodies against collagen II (10 μg/mL, DSHB, II‐II6B3) or chondroitin sulfate (10 μg/mL, DSHB, 9BA12). Secondary antibody visualization was achieved using the Vectastain ABC Universal HRP Kit (PK‐6200, Vector Laboratories) and 3,3′‐diaminobenzidine (Millipore). No primary and negative control images for immunohistochemical staining are shown in Figure S1.

### 2.7 Statistical analyses

Statistical analyses were conducted in GraphPad Prism 6. Data are presented graphically as mean with SD. Data were first tested for normality using the Shapiro‐Wilk test. If the sample size of the data set was insufficient to test for normality, data were assumed to be non‐normally distributed. Statistically significant differences between groups were assessed using a one‐way ANOVA with Tukey's posthoc test or a Kruskal‐Wallis test with Dunn's posthoc test for normally and non‐normally distributed data, respectively. A two‐way, repeated measures ANOVA with Sidak's multiple comparison test was utilized to determine statistically significant differences in vertebral angle from the serial radiographs. Statistical significance was defined as *P* < .05.

## 3 RESULTS

**FIGURE 2:**
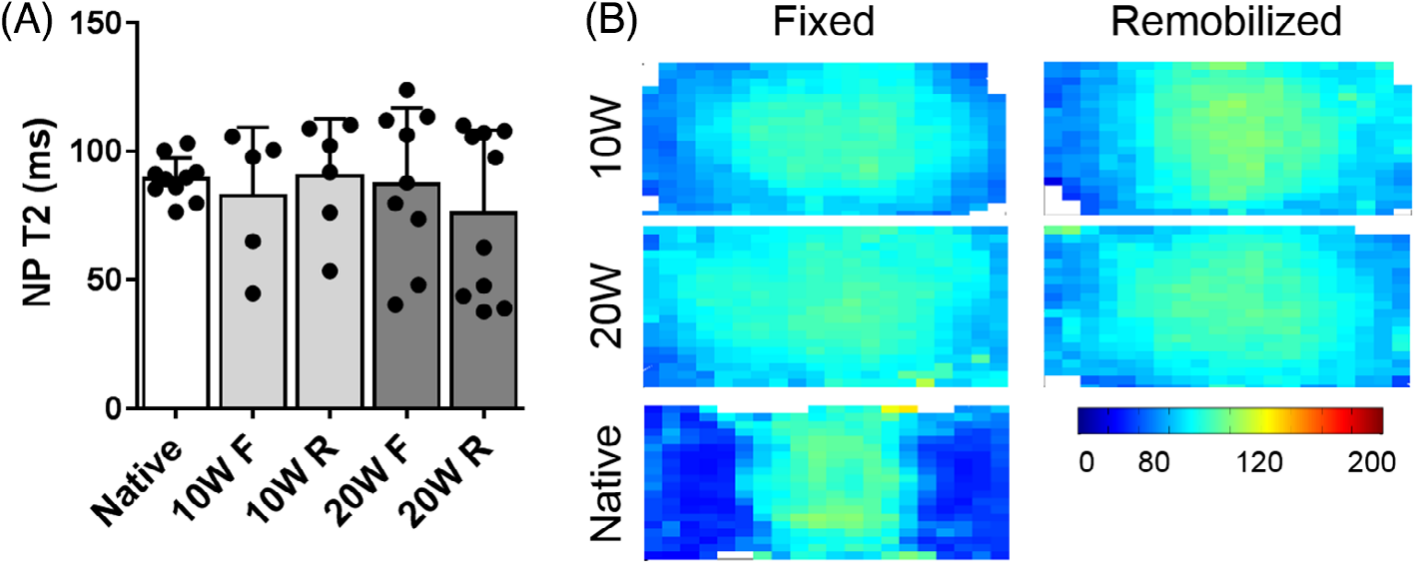
A, Quantification of the T2 relaxation time in the NP for all experimental groups compared to native controls. B, Average T2 maps for each experimental group, where the color scale represents the T2 relaxation time in milliseconds

**FIGURE 3:**
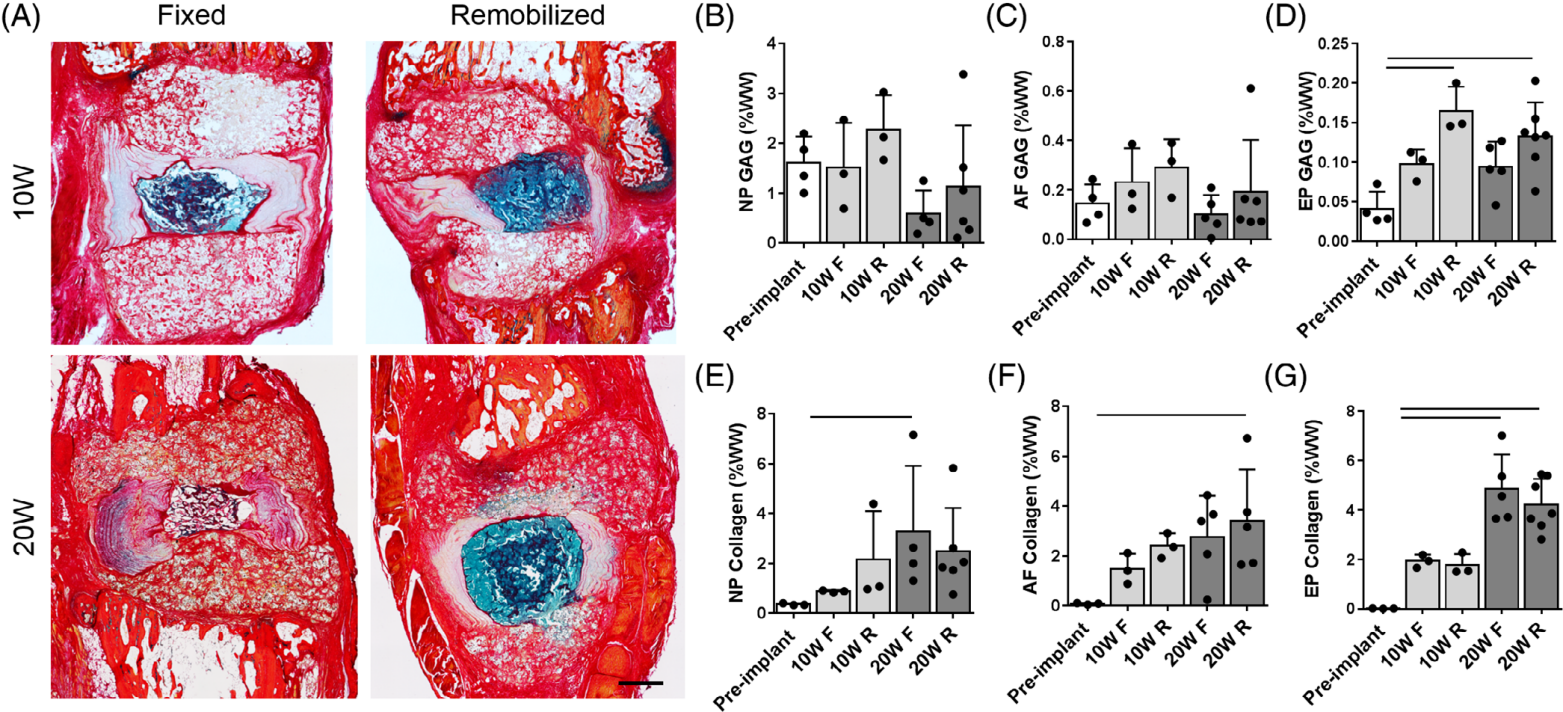
A, Representative Alcian blue and picrosirius red‐stained histology sections from each group. Scale = 1 mm. B, NP; C, AF; and D, EP proteoglycan content, in addition to, E, NP; F, AF; and G, EP collagen content, normalized to sample wet weight. Bars denote *P* < .05 between groups

**FIGURE 4:**
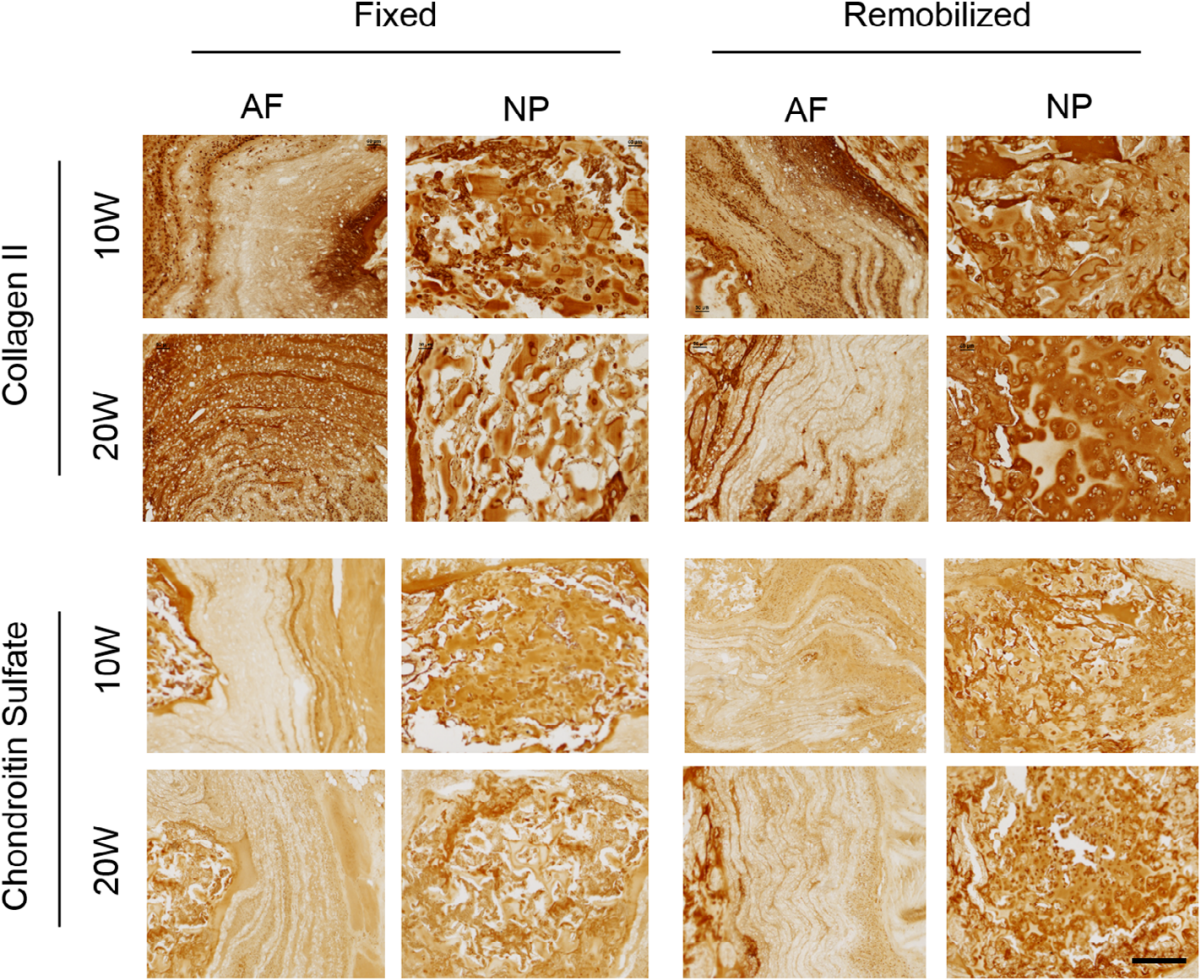
Immunohistochemical detection of collagen II and chondroitin sulfate in the AF and NP regions of fixed and remobilized groups at 10 and 20 weeks implantation. Scale = 200 μm

In all experimental groups, eDAPS maintained near native NP T2 relaxation times for to 20 weeks in vivo, with no detectable difference in NP T2 occurring following remobilization at either time point. In the disc, T2 relaxation times are correlated with both water and proteoglycan content,^28^ and in engineered discs, this is reduced with increasing deposition of matrix within the construct (Figure 2). Alcian blue and picrosirius red‐stained histology (Figure 3A) suggested a qualitative improvement in NP GAG content and reduction in collagen content following remobilization at both time points. While there were no statistically significant differences in NP or AF GAG content across groups (Figure 3B,C), GAG content of the endplate region (Figure 3D) was significantly increased from preimplantation levels in the remobilized groups at 10 (*P* = .01) and 20 (*P* = .03) weeks, as quantified via the DMMB assay. Collagen content in both the NP and AF increased with increasing duration of implantation (Figure 3E‐F). NP collagen content was significantly increased compared to preimplantation levels in only the 20 W F group (*P* = .04), while AF collagen was increased significantly in the 20 W R group only compared to preimplantation (*P* < .05). Endplate collagen was significantly increased after 20 weeks in vivo compared to preimplantation levels, with no detectable effect of remobilization (Figure 3G). Immunohistochemistry for collagen II and chondroitin sulfate (Figure 4) provided further insight into the biochemical remodeling of the eDAPS following remobilization. Collagen II and chondroitin sulfate staining in the NP region decreased from 10 to 20 weeks in the fixed groups, while staining was retained or even enhanced following remobilization, particularly at the 20‐week time point. In the AF, no appreciable differences in chondroitin sulfate staining were observed over time or following remobilization. Collagen II staining in the AF appeared to be lower in the remobilized group compared to the fixed group at the 20‐week time point.

**FIGURE 5:**
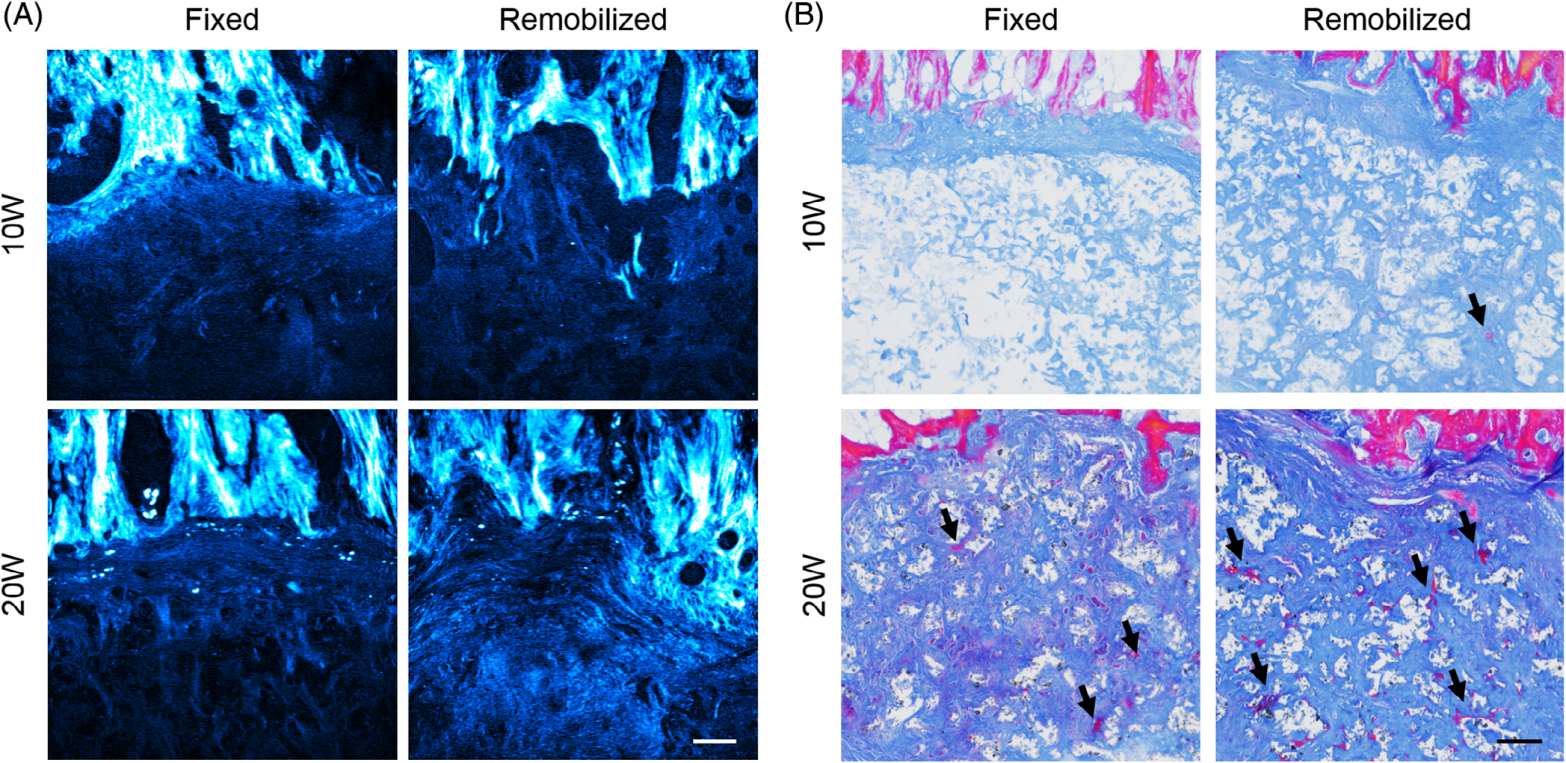
A, Representative second harmonic generation images of the PCL endplate‐vertebral body interface for each experimental group. The color scale represents collagen density and organization (light = high, dark = low). Scale = 50 μm. B, Representative histology sections stained with the Mallory Heidenhain trichrome stain (blue = unmineralized collagen, pink = mineralized collagen) from each experimental group. Scale = 200 μm

SHG imaging of the interfacial regions between the vertebral bone and PCL endplate showed alterations to the endplate region of the eDAPS following remobilization (Figure 5A). At the 10‐week time point, no appreciable differences in collagen organization or density were evident at this interface when comparing the fixed and remobilized groups. However, at 20 weeks, there was an increase in SHG signal in the PCL endplates of the remobilized group compared to the fixed group, indicating increased deposition of organized collagen at this interface. Remobilization also appeared to accelerate mineralization of this region, as evidenced by histology sections stained with the Mallory Heidenhain stain, which demonstrated increased pink staining for mineralized collagen within the PCL endplates of remobilized eDAPS compared to fixed eDAPS at both 10 and 20 weeks (Figure 5B, arrows).

**FIGURE 6:**
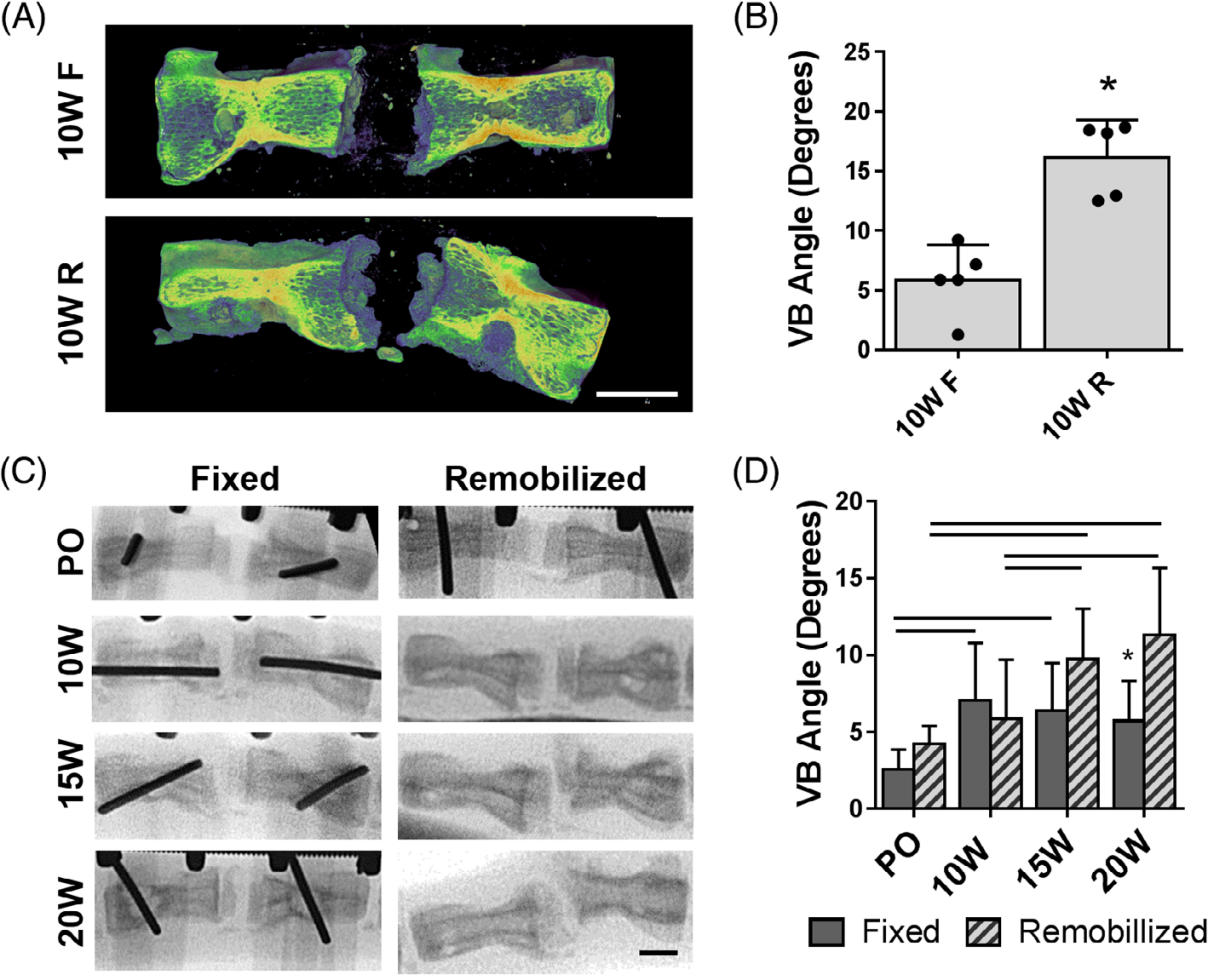
A, Representative 3D reconstructions from μCT scans demonstrating the angled morphology of the motion segment which occurs following early remobilization. (scale = 5 mm). B, Quantification of the angle between the vertebral bodies adjacent to the eDAPS implants in the 10 weeks fixed and remobilized groups. *P < .05. C, Representative radiographs of the rat caudal spine at the eDAPS implant level over the time course following remobilization up to 20 weeks (scale = 5 mm), and the D, vertebral body angle calculated at each time point in fixed and remobilized groups. Bars denote significant differences (*P* < .05) between time‐points, *P < .05 compared to the remobilized group at the same time point

Despite this evidence of improved integration of the eDAPS with the native vertebral bone, μCT and radiographic analyses revealed significant morphological alterations to whole motion segment structure. This was primarily evidenced as an angulation of the tail away from the initial incision utilized to implant the eDAPS. Quantitatively, there was a 174% increase (*P* < .001) in the angle between the vertebral bodies at 10 weeks postimplantation in the remobilized group compared to the fixed group, as measured from microCT scans (Figure 6A,B). In an effort to track this angulation phenomena following fixator removal, serial radiographs were acquired in animals in the 20 week study, where the fixator was removed at 10 weeks (Figure 6C). The angle between vertebral bodies in the remobilized group significantly increased over time (*P* < .05), while there was no detectable change in vertebral body angle in the fixed group from 10 to 20 weeks (Figure 6D). There were no differences in vertebral body angle between fixed and remobilized groups at the 10‐week time point, demonstrating that the change in vertebral body angle was not an acute phenomena due to fixator removal, but rather occurred gradually over time. At 20 weeks, the vertebral body angle was 98% higher (*P* < .001) in the remobilized group compared to the fixed group.

**FIGURE 7:**
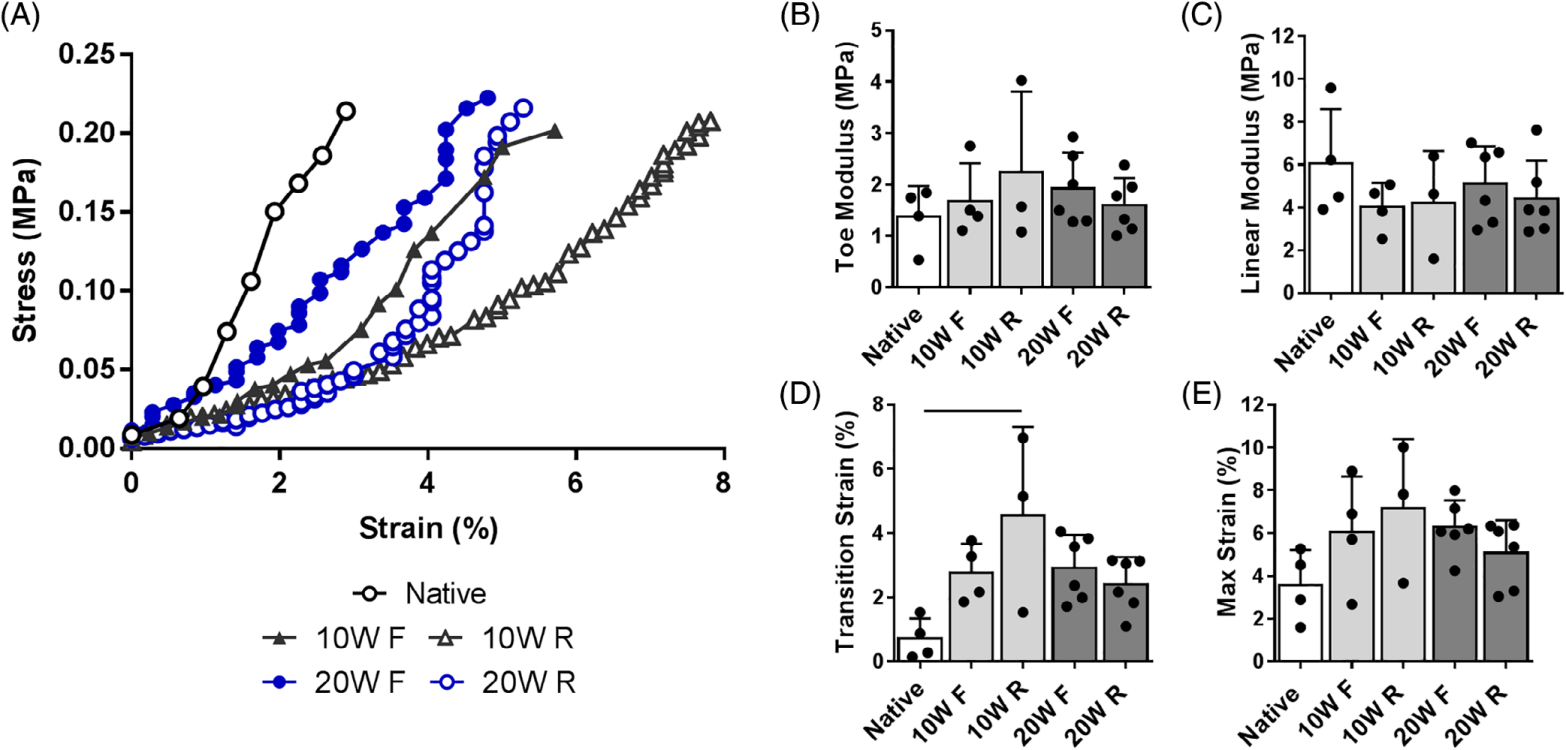
A, Representative stress‐strain curves in compression for all experimental groups compared to the native rat tail motion segment. Quantification of, B, toe and, C, linear moduli; and D, transition; and E, maximum strains in compression. Bars denote *P* < .05 between groups

**FIGURE 8:**
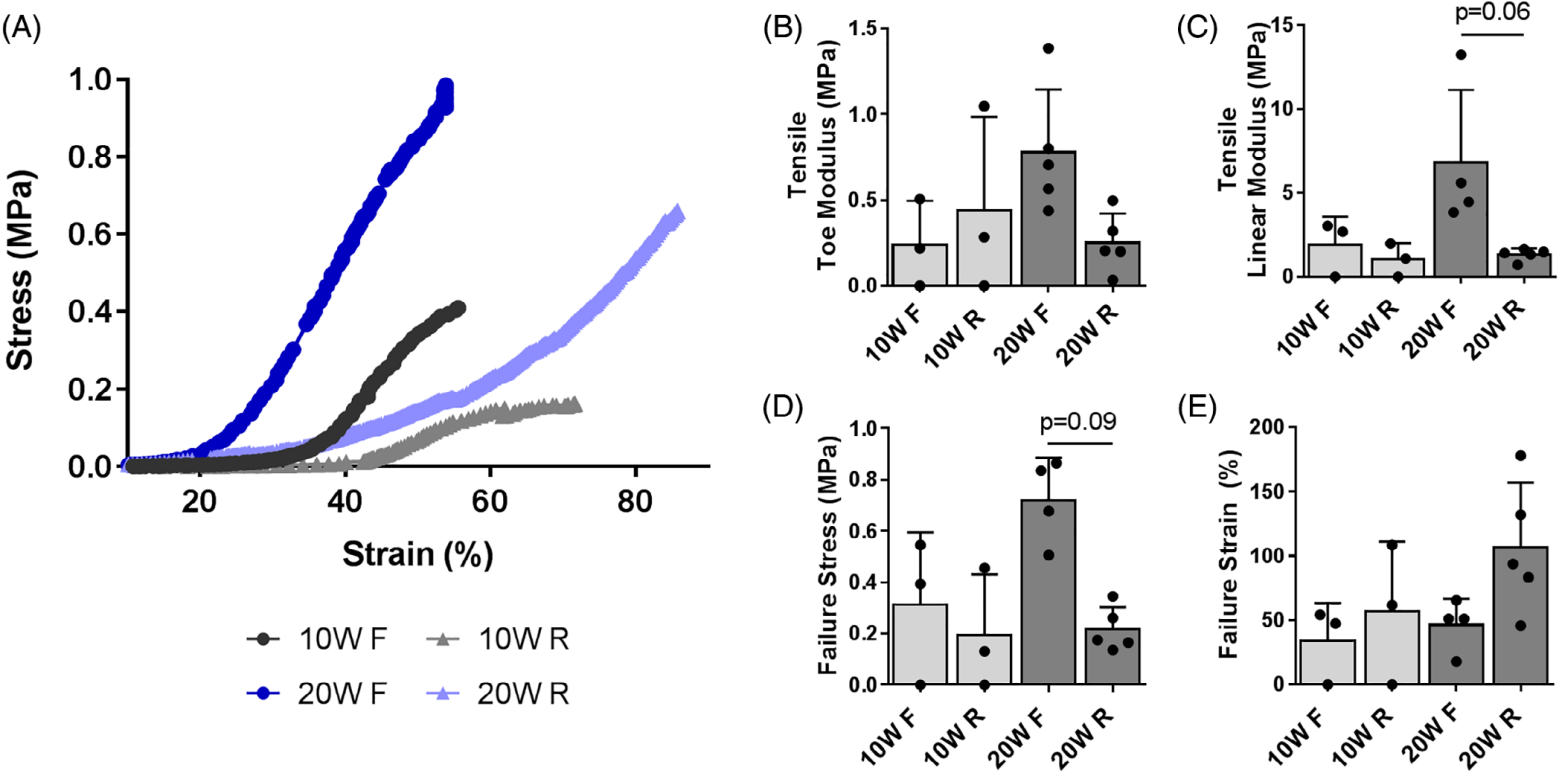
A, Representative stress–strain curves during tension to failure testing for all experimental groups. Quantification of tensile, B, toe and, C, linear moduli, and failure, D, stress and, E, strain. Bars denote trends (*P* < 0.1) between groups

In order to assess the functional consequences of the structural and compositional alterations to the eDAPS in vivo following remobilization, we performed compressive and tensile biomechanical testing of the eDAPS implanted motion segments. There were no detectable differences in compressive toe and linear modulus, or maximum strain at 3 N compression between fixed and remobilized eDAPS, or compared to native rat tail motion segments at any time point (Figure 7A‐C,E). Transition strain (Figure 7D) was significantly (*P* = .006) increased in the 10 W R group compared to native controls. Tensile strength (Figure 8A‐E) trended to be lower in the remobilized group compared to the fixed group, particularly at 20 weeks, with respect to linear modulus (mean 80% reduction) and failure stress (mean 70% reduction). In all groups, failure of the eDAPS occurred at the interface between the PCL endplate and the engineered disc, rather than at the endplate/vertebral bone interface.

## 4 DISCUSSION

Whole disc tissue engineering holds significant promise as an alternative to fusion surgery for patients with advanced disc degeneration and associated back pain. One of the challenges in the translation of tissue engineered discs is promoting the robust integration of a precultured, engineered construct with the native spinal tissues, primarily the adjacent vertebral bone. In order for this integration to occur, a period of immobilization of the motion segment is likely necessary to prevent displacement or excessive motion until such time that sufficient integration has occurred to support physiologic loading. Indeed, in animal models, immobilization has been necessary to retain tissue engineered discs within the intervertebral space.^16^, ^17^ However, long‐term immobilization or unloading is known to cause degenerative changes to the native, healthy disc,^18^, ^19^ and would likely have a detrimental effect on the composition and function of an engineered disc as well. Here, we utilize our established model of total disc replacement in a rat tail model with external fixation to investigate the effects of restoring physiologic loading (remobilization) to the implant at both early (5 weeks postimplantation) and late (10 weeks postimplantation) time points.

Our results demonstrate that remobilization was beneficial for engineered disc matrix composition, leading to improved GAG and collagen II content in the NP, and higher collagen content in the AF compared to fixed groups. These results are consistent with the large body of work studying the effects of dynamic compression on the native intervertebral disc, where physiologic loading rates and magnitudes enhance anabolic remodeling in the disc, inclusive of increased matrix deposition.^14^, ^15^, ^20^, ^32^ Additionally, dynamic loading during in vitro culture increases the GAG and collagen content of tissue engineered discs, further highlighting the importance of anabolic loading.^33^

Remobilization additionally enhanced the integration of the PCL endplate region of the eDAPS with the native vertebral body, as evidenced by increases in organized collagen content and mineralization in remobilized vs fixed groups. Mechanical loading can significantly increase bone volume formed in fracture healing models, and the timing of loading application of loading can be modulated to enhance bone vascularization as well.^34^, ^35^ This may have important implications for enhancing the remodeling of the PCL endplate region of the eDAPS, as the formation of a boney, vascularized interface will likely be critical for the long‐term maintenance of tissue engineered disc homeostasis. Taken together, these results suggest that the disc cells initially seeded within the eDAPS, and infiltrating native cells at the endplate, are capable or responding to mechanical cues in vivo.

Despite the apparent positive effects of in vivo remobilization on eDAPS composition and integration, remobilization was detrimental to motion segment morphology and mechanics, as significant angulation of the vertebral bodies at the implanted level was observed following fixator removal. This angulation still occurred, but was mitigated by prolonging the duration of immobilization (5 vs 10 weeks, 174% vs 98% increase in vertebral body angle) prior to fixator removal. The altered motion segment morphology is likely due to the disruption of the dorsal tail tendons during the eDAPS implantation surgery, leading to asymmetric loading of the motion segment following remobilization. This asymmetric loading may be particularly impactful in this implantation site, as the lack of facet joints in the rat tail allow for multi‐axial motion as the rat utilizes its tail for balance and proprioception.^36^ The biomechanical function of the eDAPS implanted motion segment in compression was largely unaffected by this altered motion segment morphology, with the exception of an increase in transition strain in the early remobilization (10 W R) group. Tensile mechanical properties, however, were impacted by remobilization, with lower tensile modulus and failure stress in the remobilized group compared to the fixed group at 20 weeks. Failure in all groups occurred at the interface between the PCL endplate and tissue engineered AF and NP components. Thus, although the integration of the PCL endplate with the vertebral body itself was enhanced with remobilization, the interface between the endplate and disc may have been weakened in the remobilized group as a consequence of the angulation of the vertebral bodies causing shearing at this interface.

Overall, the results from this study suggest that the restoration of mechanical loading to a tissue engineered disc implant in vivo will likely be essential to maintain construct homeostasis with long‐term implantation. Indeed, remobilization is a requirement for functional restoration of the spine. However, it is clear from prior studies that some degree and duration of immobilization is necessary for sufficient integration to develop and to protect against displacement of the construct. The design and optimization of internal provisional fixation systems for engineered disc replacements may be necessary to facilitate the translation of this technology toward clinical use. For example, bioresorbable plates have already been investigated ex vivo to temporarily stabilize tissue engineered disc implants,^37^ the stiffness of which could potentially be tuned to optimize bone formation within the endplate region.^38^ Finally, the detrimental effects of remobilization observed in this study, namely the alterations to motion segment morphology, are likely exacerbated in the hypermobile rat tail, which lacks stabilizing posterior elements. Future work in large animal models that have more human‐like morphology and motion, will be critical to fully understand the effects of remobilization on engineered disc structure and function.

## CONFLICT OF INTEREST

R. L. M. is a co‐editor of *JOR Spine*.

## AUTHORS’ CONTRIBUTIONS

Sarah E. Gullbrand, Harvey E. Smith, and Robert L. Mauck designed research, Sarah E. Gullbrand, Dong H. Kim, Beth G. Ashinsky, Edward D. Bonnevie, and Harvey E. Smith conducted research, Sarah E. Gullbrand, Dong H. Kim, Beth G. Ashinsky, and Edward D. Bonnevie analyzed data, Sarah E. Gullbrand, Harvey E. Smith, and Robert L. Mauck critically interpreted data, Sarah E. Gullbrand compiled the manuscript text and figures with assistance and approval from all authors. Sarah E. Gullbrand and Robert L. Mauck accept responsibility for the integrity of the data analysis.

## Supporting information


**Figure S1** (A) No primary antibody control for immunohistochemistry. (B) Full motion segment section staining for chondroitin sulfate demonstrating no positive staining in the bone adjacent to the eDAPS (negative control). Scale = 500 μm.
