# Supplementary material for: Restoration of physiologic loading modulates engineered intervertebral disc structure and function in an in vivo model
Source: JOR Spine. 2020 May 13;3(2):e1086. doi: 10.1002/jsp2.1086 (PMC7323465; doi:10.1002/jsp2.1086)
Supplement: Supplementary file 1 — Figure S1 (A) No primary antibody control for immunohistochemistry. (B) Full motion segment section staining for chondroitin sulfate demonstrating no positive staining in the bone adjacent to the eDAPS (negative control). Scale = 500 μm. [file JSP2-3-e1086-s001.docx]

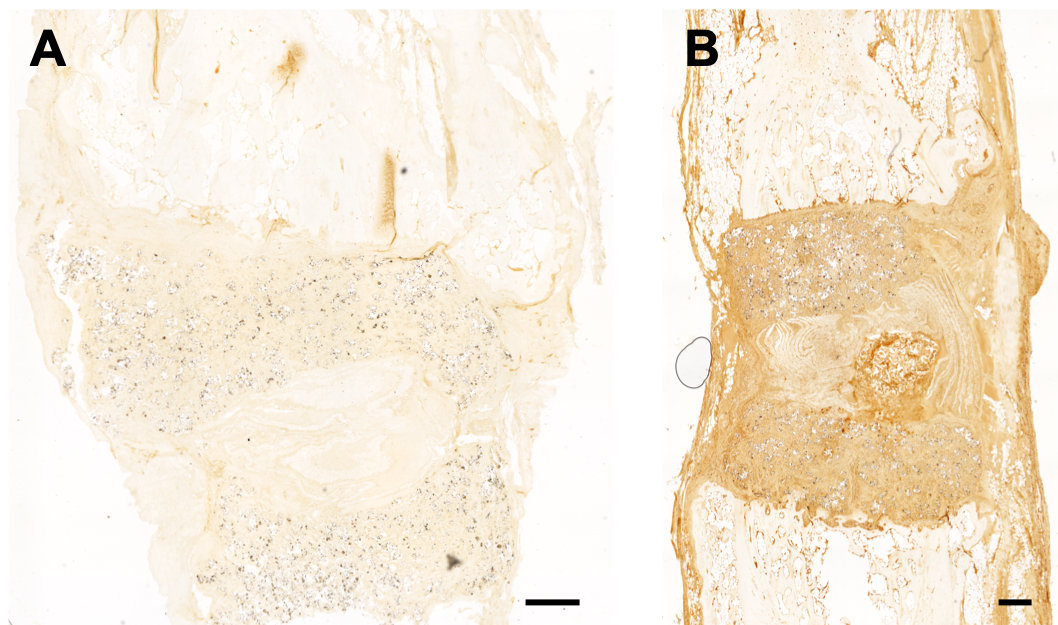


**Figure S1.** (A) No primary antibody control for immunohistochemistry. (B) Full motion segment section staining for chondroitin sulfate demonstrating no positive staining in the bone adjacent to the eDAPS (negative control). Scale = 500 µm.
